# An increase in the neutrophil-to-lymphocyte ratio during adjuvant chemotherapy indicates a poor prognosis in patients with stage II or III gastric cancer

**DOI:** 10.1186/s12885-018-5171-2

**Published:** 2018-12-17

**Authors:** Mikito Mori, Kiyohiko Shuto, Chihiro Kosugi, Kazuo Narushima, Hideki Hayashi, Hisahiro Matsubara, Keiji Koda

**Affiliations:** 1 Department of Surgery, Teikyo University Chiba Medical Center, 3426-3 Anesaki, Ichihara, Chiba, 299-0111 Japan; 2 Center for Frontier Medical Engineering, Chiba University, Chiba, Japan; 3 Department of Frontier Surgery, Graduate School of Medical and Pharmaceutical Science, Chiba University, Chiba, Japan

**Keywords:** Gastric cancer, Neutrophil-to-lymphocyte ratio, Platelet-to-lymphocyte ratio, Adjuvant chemotherapy, Overall survival, Relapse-free survival

## Abstract

**Background:**

The neutrophil-to-lymphocyte ratio (NLR) and the platelet-to-lymphocyte ratio (PLR) are associated with poor prognoses in patients with gastric cancer; however, few studies have focused on the dynamic changes in these ratios during the treatment of patients with gastric cancer. Here, we assessed the clinical utility of changes in these ratios as prognostic indicators in patients with stage II or III gastric cancer who received adjuvant chemotherapy.

**Methods:**

We retrospectively reviewed 100 patients who received S-1 adjuvant chemotherapy at ≥70% of the relative dose intensity, and their NLRs and PLRs were evaluated at different times: prior to gastrectomy and upon commencement and termination of adjuvant chemotherapy. To assure the clinical utility of the changes in NLR and PLR as prognostic indicators, other clinical factors were assessed as well.

**Results:**

Disease recurred in 35 patients as follows: lymph node metastasis (17 patients, 17.0%), peritoneal metastasis (12 patients, 12.0%), and hematogenous metastasis (6 patients, 6.0%); 24 patients died. An increase in the NLR during adjuvant chemotherapy with S-1 was identified as an independent indicator associated with overall survival (hazard ratio [HR] 6.736, 95% confidence interval [CI] 2.420–18.748; *P* < 0.001), and relapse-free survival (HR 5.309, 95% CI 2.585–10.901; *P* < 0.001).

**Conclusion:**

An increase in the NLR during S-1 adjuvant chemotherapy may be a useful prognostic indicator in patients with stage II or III gastric cancer.

## Background

Gastric cancer (GC) is the fifth most common malignancy and the third leading cause of cancer-related death worldwide. Although the incidence of GC has declined in East Asia, GC is still the second most common cancer in Eastern Asia, including Japan [1]. In Japan, curative gastrectomy with D2 lymph-node dissection in patients with stage II or III GC is the main treatment for GC, and adjuvant therapy is also required to improve overall survival (OS) and relapse-free survival (RFS) [2].

Despite the effectiveness of adjuvant chemotherapy with S-1, further improvements in the treatments for patients with stage III GC are required, and approaches such as the development of combination chemotherapy [3, 4] and the analyses of molecular mechanisms [5] have been conducted. Several studies have focused on the relationship between systemic inflammation and cancer progression [6], and diverse prognostic scores based on systemic inflammatory variables have been assessed to predict prognosis in patients with cancer. In particular, the neutrophil-to-lymphocyte ratio (NLR) and platelet-to-lymphocyte ratio (PLR) are useful for predicting prognosis in patients with certain malignancies [7–9]. NLR and PLR are considered useful predictors of survival in patients with GC [10–12]. However, most of studies emphasize the importance of the pretreatment NLR and PLR for GC, and few reports discuss the importance of the change in the NLR and PLR after treatment [13–15]. To assess how the changes in NLR and PLR reflected prognoses of patents with stage II or III GC who received adjuvant chemotherapy, we investigated the relationship between clinical factors such as change in the NLR or PLR during adjuvant chemotherapy and the survival of patients with stage II or III GC.

## Methods

### Patients

One hundred and eighteen patients were histologically diagnosed with stage II or III GC after curative gastrectomy with D2 lymph-node dissection between January 2006 and January 2017 at Teikyo University Chiba Medical Center and Chiba University Hospital. Pathological staging was performed according to the cancer staging system for GC recommended in the 8th edition of the American Joint Committee on Cancer (AJCC) Cancer Staging Manual. In the analysis, there were no patients who died within 30 days after surgery, died of causes unrelated to cancer, had other malignancies, or had inflammatory diseases. To eliminate potential effects on relapse and survival, 18 out of 118 patients were excluded for the following reasons: 10 patients treated with neoadjuvant chemotherapy and 8 patients treated with S-1 adjuvant chemotherapy at less than 70% of the relative dose intensity (RDI). Therefore, we retrospectively reviewed 100 patients who received S-1 adjuvant chemotherapy at greater than 70% of the RDI for 1 year or until tumor recurrence.

### Treatment

After curative gastrectomy, all patients received adjuvant chemotherapy with S-1 (TS-1, Taiho Pharmaceutical, Tokyo, Japan), which is an orally active preparation combining tegafur, gimeracil, and oteracil in a molar ratio of 1:0.4:1. S-1 (80–120 mg per day) was administered for 4 weeks followed by a 2-week rest or for 2 weeks followed by a 1-week rest. The daily dose of S-1 was determined based on body surface area. This 3- or 6-week cycle was repeated for 1 year or until a tumor recurrence was objectively diagnosed.

### Evaluation of the NLR and PLR

A routine blood examination was performed before curative gastrectomy and during adjuvant chemotherapy. The NLR or PLR was calculated by dividing the lymphocyte count into neutrophil or platelet count. The pNLR, iNLR, and fNLR (NLRs), were defined as follows: the preoperative NLR (pNLR), the NLR on the initial day of adjuvant chemotherapy divided by the preoperative NLR (iNLR), and the NLR on the final day of adjuvant chemotherapy divided by the NLR on the initial day of adjuvant chemotherapy (fNLR), respectively. The pPLR, iPLR, and fPLR (PLRs) were similarly defined. Patients were divided into two groups according to a cutoff value. For the pNLR and pPLR, the median was defined as the cutoff value. The patient was classified as positive pNLR or positive pPLR when the pNLR or pPLR was greater than the median (pNLR or pPLR ≥ the median). The patient was classified as negative pNLR or negative pPLR when the pNLR or pPLR was less than the median (pNLR or pPLR < the median). One was defined as the cutoff value for the other NLRs and PLRs such as iNLR, iPLR, fNLR, and fPLR. Furthermore, the patient was classified as positive NLR or positive PLR when the NLR or PLR was ≥1 (NLR or PLR ≥1). The patient was classified as negative NLR or negative PLR when the NLR or PLR was < 1 (NLR or PLR < 1).

### Statistical analysis

The relationships between clinical factors and NLRs or PLRs were analyzed using Fisher’s exact test. OS and RFS curves were generated using the Kaplan–Meier method, and univariate analysis of survival was performed using the log-rank test. Multivariate analysis was performed using a Cox proportional-hazards model to determine the statistical significance of prognostic factors. *P* values in multiple comparisons were corrected using a false discovery rate. All statistical analyses were performed using SPSS for Windows (version 20.0, IBM Corp., Armonk, NY, USA).

## Results

The clinical characteristics of 100 patients (27 women and 73 men) with stage II or III GC who received adjuvant chemotherapy with S-1 are summarized in Table 1. The median age was 66 years (range, 36–82 years), including 41 patients < 65 years and 59 patients ≥65 years. The median tumor size was 60 mm (range, 15–170 mm), including 48 patients with tumors < 60 mm and 52 patients with tumors ≥60 mm. The tumor cells of 35 and 65 patients were histologically classified as differentiated and undifferentiated, respectively. Pathological tumor (pT) stages were as follows: 5 patients, pT1; 12 patients, pT2; 41 patients, pT3; and 42 patients, pT4. Pathological nodal (pN) stages were as follows: 14 patients, pN0; 25 patients, pN1; 31 patients, pN2; and 30 patients, pN3. Thirty-nine patients were diagnosed with pathological cancer stage (pStage) II GC and 61 patients were diagnosed with pStage III GC.

**Table 1:** Demographics of GC patients treated with S-1 adjuvant chemotherapy

Factors	*N* = 100
Sex (M/F)	73 / 27
Age (< 65/≥65 years)	41 / 59
Tumor size (< 60/≥60 mm)	48 / 52
Histologic type (Diff/Undiff)	35 / 65
pT (1/2/3/4)	5 / 12 / 41 / 42
pN (0/1/2/3)	14 / 25 / 31 / 30
pStage (II/III)	39 / 61
Lymphatic invasion (+/−)	80 / 20
Venous invasion (+/−)	78 / 22
pNLR (+/−)	50 / 50
iNLR (+/−)	26 / 74
fNLR (+/−)	38 / 62
pPLR (+/−)	50 / 50
iPLR (+/−)	50 / 50
fPLR (+/−)	35 / 65
Recurrence (+/−)	35 / 65
Site of relapse (H/P/LYM/Lo)	6 / 12 / 17 / 2
Outcome (D/A)	24 / 76

M, male; F, female; Diff, differentiated type; Undiff, undifferentiated type; pT, pN, pStage = pathological T stage, N stage. Pathological cancer stage according to the 8th edition of the American Joint Committee on Cancer (AJCC) Cancer Staging Manual; pNLR or pPLR, preoperative neutrophil or platelet-to-lymphocyte ratio; iNLR and iPLR, the ratio of the NLR or PLR on the initial day of adjuvant chemotherapy to the pNLR or pPLR; fNLR and fPLR, the ratio of the NLR or PLR on the final day of adjuvant chemotherapy to the iNLR or iPLR; H, hematogenous metastasis; P, peritoneal metastasis; LYM, lymph node metastasis; Lo, local recurrence; D/A, dead or alive

The median pNLR was 2.6 (range, 0.8–9.8), and the median pPLR was 149.4 (range, 67.7–555.3). Fifty patients were classified as positive pNLR or pPLR, and 50 patients were classified as negative pNLR or pPLR. Thirty-eight and 62 patients were classified as positive fNLR and negative fNLR, respectively. Thirty-five and 65 patients were classified as positive and negative fPLR, respectively. Thirty-five patients developed recurrences as follows: lymph node metastasis (17 patients, 17.0%), peritoneal metastasis (12 patients, 12.0%), hematogenous metastasis (6 patients, 6.0%), and local recurrence (2 patients, 2%). Twenty-four patients died of GC during the median follow-up period of 37.1 months (range, 5.3–108.8 months).

To evaluate whether the NLR or PLR may serve as a useful indicator of OS and RFS, all NLRs and PLRs were assessed using receiver operating characteristic (ROC) curves. In the analysis of OS, the area under the curves (AUCs) of pNLR, iNLR, and fNLR were 0.659 (sensitivity, 70.8%; specificity, 56.6%), 0.463 (sensitivity, 33.3%; specificity, 76.3%), and 0.748 (sensitivity, 75.0%; specificity, 69.7%), respectively. The AUCs of pPLR, iPLR, and fPLR were 0.666 (sensitivity, 66.7%; specificity, 55.3%), 0.446 (sensitivity, 41.7%; specificity, 47.4%), and 0.708 (sensitivity, 58.3%; specificity, 72.4%), respectively. For RFS, the AUCs of pNLR, iNLR, and fNLR were 0.619 (sensitivity, 62.9%; specificity, 56.9%), 0.453 (sensitivity, 25.7%; specificity, 73.8%), and 0.706 (sensitivity, 68.6%; specificity, 73.8%), respectively. The AUCs of pPLR, iPLR, and fPLR were 0.605 (sensitivity, 54.3%; specificity, 52.3%), 0.447 (sensitivity, 40.0%; specificity, 44.6%), and 0.603 (sensitivity, 42.9%; specificity, 69.2%), respectively.

**Fig. 1:**
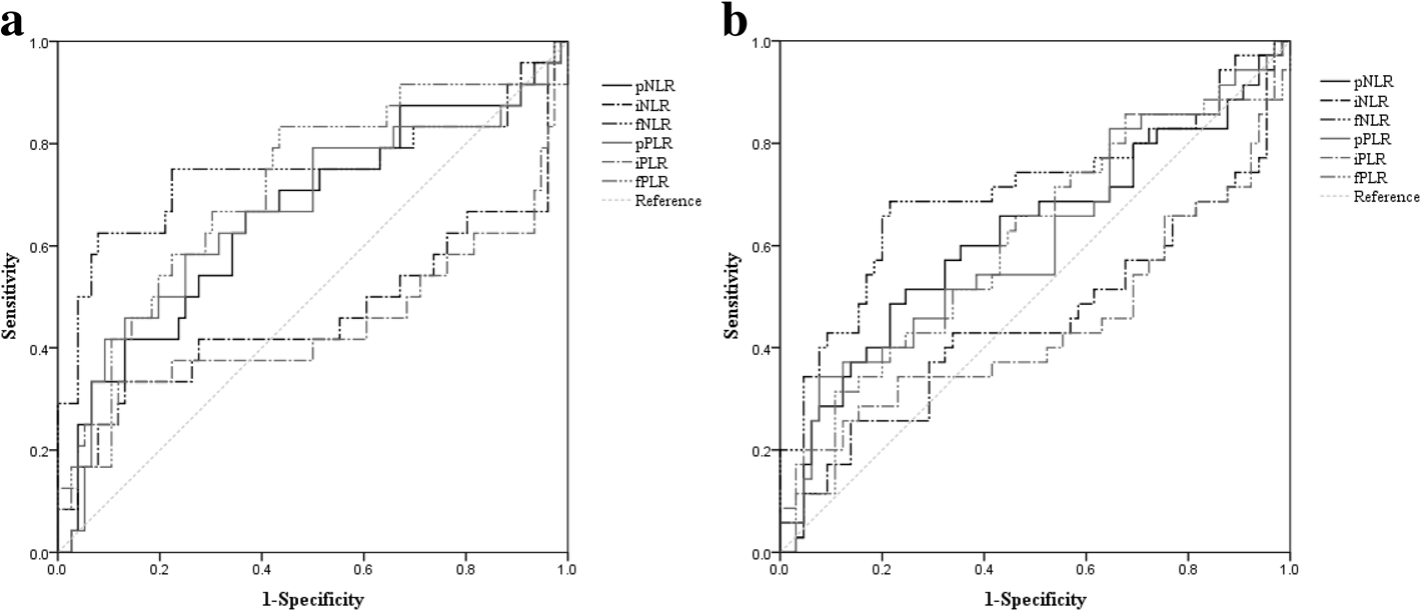
Evaluation of NLRs and PLRs as prognostic indicators using receiver operating characteristic curves. **a** overall survival, **b** relapse-free survival

The ROC curves suggested that fNLR was the best prognostic indicator of NLRs and PLRs (Fig. 1). There was no significant difference between positive and negative pNLRs or iNLRs. In contrast, there was a significant difference in recurrences and outcomes between positive and negative fNLRs (Table 2). There was no significant difference between positive and negative iPLRs or fPLRs. However, there was a significant difference between positive and negative pPLRs associated with pT and pStage (Table 3). Univariate analysis of OS revealed that tumor size (*P* = 0.008), histological type (*P* = 0.005), fNLR (*P* < 0.001), and fPLR (*P* = 0.004) were associated with a shorter OS.

**Table 2:** Relationship between NLRs and clinical factors

Factor	pNLR (+)	pNLR (−)	*P-value*	iNLR (+)	iNLR (−)	*P-value*	fNLR (+)	fNLR (−)	*P-value*
Sex (M/F)	34/16	39/11	0.368	23/ 3	50/24	0.043	28/10	45/17	1.000
Age (< 65/≥65 years)	21/29	20/30	1.000	11/15	30/44	0.875	16/22	25/37	1.000
Tumor size (< 60/≥60 mm)	20/30	28/22	0.161	14/12	34/40	0.504	16/22	32/30	0.412
Histologic type (Diff/Undiff)	15/35	20/30	0.402	8/18	27/47	0.644	11/27	24/38	0.390
pT (1/2/3/4)	1/ 6/18/25	4/ 6/23/17	0.275	2/ 4/10/10	3/ 8/31/32	0.731	2/ 7/11/18	3/ 5/30/24	0.180
pN (0/1/2/3)	4/11/14/21	10/14/17/ 9	0.045	2/11/ 9/ 4	12/14/22/26	0.056	3/ 9/14/12	11/16/17/18	0.510
pStage (II/III)	13/37	26/24	0.013	12/14	27/47	0.484	15/23	24/38	1.000
Lymphatic invasion (+/−)	43/ 7	37/13	0.211	21/ 5	59/15	1.000	28/10	52/10	0.303
Venous invasion (+/−)	40/10	38/12	0.810	20/ 6	58/16	1.000	30/ 8	48/14	1.000
Recurrence (+/−)	22/28	13/37	0.093	9/17	26/48	1.000	24/14	11/51	< 0.001*
Outcome (D/A)	17/33	7/43	0.034	8/18	16/58	0.425	18/20	6/56	< 0.001*

M, male; F, female; Diff, differentiated type; Undiff, undifferentiated type; pT, pN, pStage = pathological T stage, N stage. Pathological cancer stage according to the 8th edition of the American Joint Committee on Cancer (AJCC) Cancer Staging Manual; pNLR, preoperative neutrophil-to-lymphocyte ratio; iNLR, the ratio of the NLR on the initial day of adjuvant chemotherapy to the pNLR; fNLR, the ratio of the NLR on the final day of adjuvant chemotherapy to the iNLR; D/A, dead or alive; *P-value indicates statistical significance after false discovery rate correction

**Table 3:** Relationship between PLRs and clinical factors

Factor	pPLR (+)	pPLR (−)	*P-value*	iPLR (+)	iPLR (−)	*P-value*	fPLR (+)	fPLR (−)	*P-value*
Sex(M/F)	32/18	41/ 9	0.070	39/11	34/16	0.368	24/11	49/16	0.486
Age (< 65/≥65 years)	24/26	17/33	0.222	21/29	20/30	1.000	15/20	26/39	0.833
Tumor size (< 60/≥60 mm)	21/29	27/23	0.317	26/24	22/28	0.548	15/20	33/32	0.531
Histologic type (Diff/Undiff)	15/35	20/30	0.402	19/31	16/34	0.675	8/27	27/38	0.080
pT (1/2/3/4)	0/ 6/16/28	5/ 6/25/14	0.006*	4/ 8/21/17	1/ 4/20/25	0.210	3/ 5/11/16	2/ 7/30/26	0.393
pN (0/1/2/3)	4/13/12/21	10/12/19/ 9	0.030	8/15/16/11	6/10/15/19	0.332	4/ 6/13/12	10/19/18/18	0.480
pStage (II/III)	12/38	27/23	0.004*	25/25	14/36	0.040	13/22	26/39	0.832
Lymphatic invasion (+/−)	42/ 8	38/12	0.454	39/11	41/ 9	0.803	26/ 9	54/11	0.307
Venous invasion (+/−)	39/11	39/11	1.000	38/12	40/10	0.810	25/10	53/12	0.312
Recurrence (+/−)	19/31	16/34	0.675	14/36	21/29	0.208	15/20	20/45	0.274
Outcome (D/A)	16/34	8/42	0.100	10/40	14/36	0.483	14/21	10/55	0.013

M, male; F, female; Diff, differentiated type; Undiff, undifferentiated type; pT, pN, pStage = pathological T stage, N stage. Pathological cancer stage according to the 8th edition of the American Joint Committee on Cancer (AJCC) Cancer Staging Manual; pPLR, preoperative platelet-to-lymphocyte ratio; iPLR, the ratio of the PLR on the initial day of adjuvant chemotherapy to pPLR; fPLR, the ratio of the PLR on the final day of adjuvant chemotherapy to iPLR; D/A, dead or alive; *P-value indicates statistical significance after false discovery rate correction

**Fig. 2:**
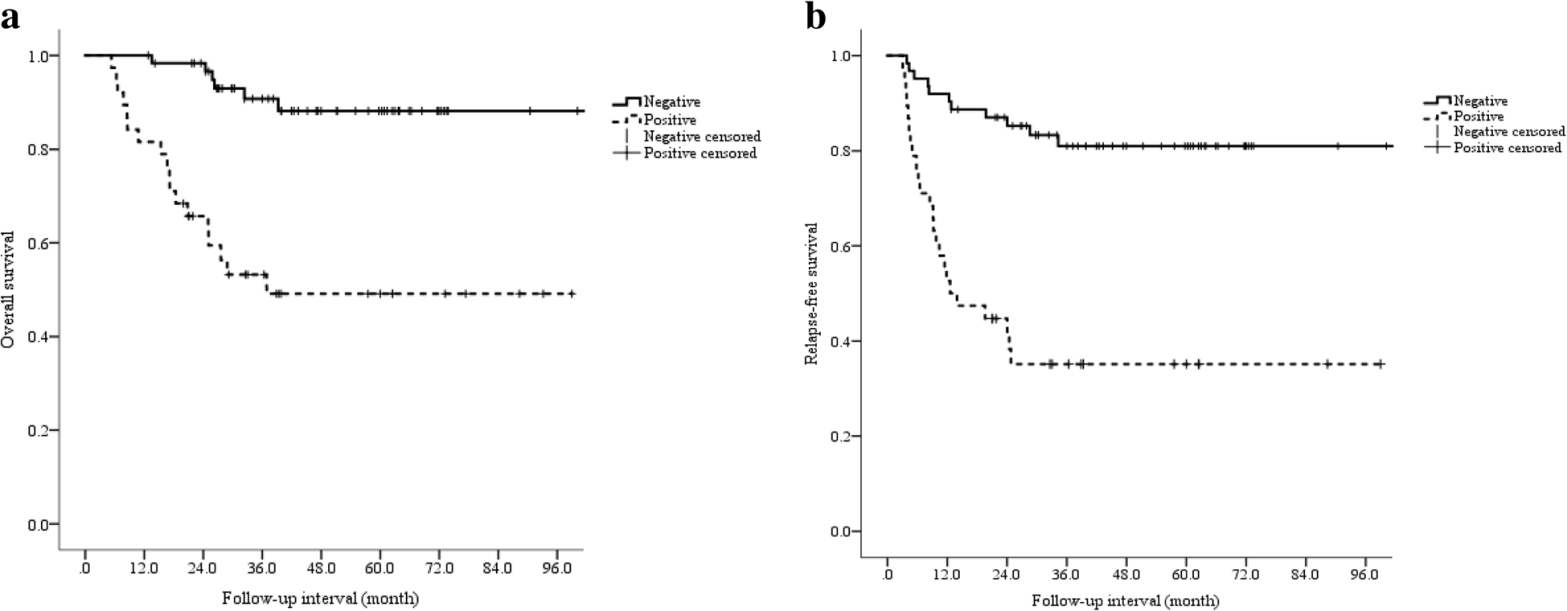
Survival curves of positive and negative fNLR values using the log-rank test. **a** overall survival (*P* < 0.001), **b** relapse-free survival (*P* < 0.001)

Multivariate analysis of the significant variables identified using univariate analysis of OS revealed that tumor size (HR 3.115, 95% CI 1.230–7.889; *P* = 0.017), histological type (HR 4.472, 95% CI 1.308–15.287; *P* = 0.017) and fNLR (HR 6.736, 95% CI 2.420–18.748; *P* < 0.001) were independently associated with a shorter OS, and the fNLR was identified as a significant indicator of OS (Table 4). In univariate analysis of RFS, fNLR (*P* < 0.001) was only associated with a shorter RFS. Multivariate analysis of the significant variables identified by univariate analysis of RFS revealed that fNLR (HR 5.309, 95% CI 2.585–10.901; *P* < 0.001) was independently associated with a shorter RFS, and fNLR was identified as a significant indicator of RFS (Table 5). We suggest, therefore, that the fNLR may be the best prognostic value for patients during adjuvant chemotherapy with S-1 (Fig. 2).

**Table 4:** Relationship between clinical factors and OS in GC patients treated with S-1 adjuvant chemotherapy

Factors	*N* = 100	Univariate analysis	Multivariate analysis	
		*P-valuea*	HR (95% CI)b	*P-valueb*
Sex (M/F)	73/27	0.417		
Age (< 65/≥65 years)	41/59	0.558		
Tumor size (< 60/≥60 mm)	48/52	0.008*	3.115 (1.230–7.889)	0.017
Histologic type (Diff/Undiff)	35/65	0.005*	4.472 (1.308–15.287)	0.017
pT (1/2/3/4)	5/12/41/42	0.366		
pN (0/1/2/3)	14/25/31/30	0.023		
pStage (II/III)	39/61	0.043		
pNLR (+/−)	50/50	0.018		
pPLR (+/−)	50/50	0.074		
iNLR (+/−)	26/74	0.455		
iPLR (+/−)	50/50	0.308		
fNLR (+/−)	38/62	< 0.001*	6.736 (2.420–18.748)	< 0.001
fPLR (+/−)	35/65	0.004*		
CEA (< 5.0/≥5.0 ng/ml)	26/74	0.118		
CA19–9 (< 37.0/≥37.0 U/ml)	18/82	0.262		

M, male; F, female; Diff, differentiated type; Undiff, undifferentiated type; pT, pN, pStage = pathological T stage, N stage. Pathological cancer stage according to the 8th edition of the American Joint Committee on Cancer (AJCC) Cancer Staging Manual; pNLR or pPLR, preoperative neutrophil or platelet-to-lymphocyte ratio; iNLR and iPLR, the ratio of the NLR or PLR on the initial day of adjuvant chemotherapy to the pNLR or pPLR; fNLR and fPLR, the ratio of the NLR or PLR on the final day of adjuvant chemotherapy to the iNLR or iPLR; CEA, carcinoembryonic antigen; CA19–9, carbohydrate antigen 19–9; ^a^Log-rank test; ^b^Cox proportional hazards model; *P-value indicates statistical significance after false discovery rate correction

**Table 5:** Relationship between clinical factors and RFS in GC patients treated with S-1 adjuvant chemotherapy

Factors	*N* = 100	Univariate analysis	Multivariate analysis	
		*P-valuea*	HR (95% CI)b	*P-valueb*
Sex (M/F)	73/27	0.821		
Age (< 65/≥65 years)	41/59	0.558		
Tumor size (< 60/≥60 mm)	48/52	0.093		
Histologic type (Diff/Undiff)	35/65	0.199		
pT (1/2/3/4)	5/12/41/42	0.226		
pN (0/1/2/3)	14/25/31/30	0.014		
pStage (II/III)	39/61	0.016		
pNLR (+/−)	50/50	0.057		
pPLR (+/−)	50/50	0.494		
iNLR (+/−)	26/74	0.965		
iPLR (+/−)	50/50	0.204		
fNLR (+/−)	38/62	< 0.001*	5.309 (2.585–10.901)	< 0.001
fPLR (+/−)	35/65	0.144		
CEA (< 5.0/≥5.0 ng/ml)	26/74	0.262		
CA19–9 (< 37.0/≥37.0 U/ml)	18/82	0.055		

M, male; F, female; Diff, differentiated type; Undiff undifferentiated type; pT, pN, pStage = pathological T stage, N stage. Pathological cancer stage according to the 8th edition of the American Joint Committee on Cancer (AJCC) Cancer Staging Manual; pNLR or pPLR, preoperative neutrophil or platelet-to-lymphocyte ratio; iNLR and iPLR, the ratio of the NLR or PLR on the initial day of adjuvant chemotherapy to the pNLR or pPLR; fNLR and fPLR, the ratio of the NLR or PLR on the final day of adjuvant chemotherapy to the iNLR or iPLR; CEA, carcinoembryonic antigen; CA19–9, carbohydrate antigen 19–9; ^a^Log-rank test; ^b^Cox proportional hazards model; *P-value indicates statistical significance after false discovery rate correction

## Discussion

There is a significant relationship between inflammation and cancer, as hypothesized by Rudolf Virchow [16]. The relationship between the NLR and survival is complicated, and the precise mechanisms are unknown. Generally, neutrophils are the most common leukocyte subset in human peripheral blood, accounting for 50–70% of total circulating leukocytes. Furthermore, neutrophils are considered essential for protecting the host and for the development of cancer-associated inflammation, because neutrophils are thought to release cytokines, chemokines, and granule proteins, which produce a favorable microenvironment for tumor growth and promote tumor progression [6, 17]. In contrast, lymphocytes play a vital role in suppressing tumor development, and the diverse functions of lymphocytes may be related to protection against the development and progression of cancer [18, 19].

Based on the inverse relationship between neutrophils and lymphocytes, the NLR is considered to provide useful information related to cancer progression. A representative study assessing the relationship between pretreatment NLRs and survival in 1028 patients with primary GC who underwent gastrectomy was performed by Shimada et al. Their data suggested that a high preoperative NLR serves as an independent risk factor for OS [11]. Jung et al. evaluated 293 patients who had undergone curative gastrectomy. Their analysis established that a high preoperative NLR was significantly related to a poor OS or disease-free survival in patients with stage III or IV GC [12].

With advances in chemotherapy, several studies have focused on pretreatment NLRs and PLRs as useful predictors of response to chemotherapy in patients with certain malignancies [20–22]. In the analysis of pretreatment NLRs or PLRs, an important potential limitation was that cutoff values for NLRs or PLRs differed among those studies, although their results indicated a significant association between high blood-neutrophil counts and poor clinical outcomes in GC.

Studies focused on the dynamic changes in the NLR after treatment showed that the change in the NLR in patients with renal cell carcinoma was associated with outcomes and clinicopathological parameters [23, 24]. Moreover, the change in the NLR was a more statistically robust predictor of OS in patients with non-small cell lung cancer or urothelial carcinoma compared with the pretreatment NLR [25, 26]. Although most studies in GC have focused on pretreatment NLRs or PLRs, few reports have focused on the dynamic change in the NLR or PLR after treatment in GC [13–15]. Indeed, few studies have focused on the change in the NLR during chemotherapy administered to patients with advanced GC. For example, Lee et al. found that the NLR, PLR, and changes in their values served as independent prognostic indicators of OS in patients with unresectable and recurrent GC who received FOLFOX chemotherapy [13]. Jin et al. suggested that the NLR was a potential predictor of survival in patients with stage III or IV GC who received neoadjuvant chemotherapy [14]. Therefore, the change in the NLR associated with treatment was a more meaningful measurement than that provided by the pretreatment NLR, because the change in the NLR may reflect a dynamic reaction of the immune response caused by the treatment.

We selectively analyzed 100 patients who received S-1 adjuvant chemotherapy at greater than 70% of the RDI. In view of the RDI, some studies have demonstrated that insufficient RDI of chemotherapy was related to a poor prognosis in some malignancies such as breast, ovarian, colon, and pancreatic cancers [27–30]. In GC, two studies suggested that a decreased RDI of S-1 will lessen the efficacy of S-1 adjuvant chemotherapy for GC and may lead to a poor prognosis [31, 32]. Our present study included only patients with stage II or III GC who received sufficient adjuvant chemotherapy with S-1 after surgery and excluded patients with very advanced metastatic disease. Furthermore, the effect on neoadjuvant chemoradiation therapy may bias the results. Although our current findings should be interpreted with caution because we performed a retrospective analysis of a small number of patients, and additional assessments are required to elucidate the relationship between the NLR and systemic inflammatory or immune responses at the time of recurrence, we believe that its potential clinical significance justifies further investigation.

## Conclusions

In conclusion, our study demonstrated that fNLR was a better prognostic indicator compared with pNLR in patients with stage II or III GC who received sufficient adjuvant chemotherapy with S-1. The change in the NLR during adjuvant chemotherapy with S-1 may be easier to determine, less expensive to measure, and useful for indicating a poor prognosis in patients with stage II or III GC.

## Abbreviations

AUC: Area under the ROC curve
fNLR: The ratio of the NLR on the final day of adjuvant chemotherapy to the NLR on the initial day of adjuvant chemotherapy
fPLR: The ratio of the PLR on the final day of adjuvant chemotherapy to the PLR on the initial day of adjuvant chemotherapy
GC: Gastric cancer
iNLR: The ratio of the NLR on the initial day of adjuvant chemotherapy to the preoperative NLR
iPLR: The ratio of the PLR on the initial day of adjuvant chemotherapy to the preoperative PLR
NLR: Neutrophil-to-lymphocyte ratio
OS: Overall survival
PLR: Platelet-to-lymphocyte ratio
pNLR: Preoperative NLR
pPLR: Preoperative PLR
RFS: Relapse-free survival
ROC: Receiver operating characteristics
